# Emergence of Microvariants of African Swine Fever Virus Genotype II in the Asia–Pacific

**DOI:** 10.1155/tbed/9990044

**Published:** 2025-06-20

**Authors:** James O'Dwyer, Hung Vo Van, Nguyen Thanh Phuong, Patrick Mileto, Orlando Mercado, Felisiano da Conceição, Joanita Bendita da Costa Jong, Ilagi Puana, Matthew J. Neave, David T. Williams

**Affiliations:** ^1^Australian Centre for Disease Preparedness, Commonwealth Scientific and Industrial Research Organisation, Geelong, Australia; ^2^Department of Animal Health, Regional Animal Health Office No. 6, Ho Chi Minh City, Vietnam; ^3^National Animal Health and Food Testing Laboratory, National Animal and Quarantine Inspection Authority, Port Moresby, Papua New Guinea; ^4^National Directorate of Veterinary Services of the Ministry of Agriculture and Fisheries, Government of Timor-Leste, Comoro, East Timor

**Keywords:** African swine fever, ASFV Genotype II, genetic epidemiology, microevolution, whole-genome sequencing

## Abstract

African swine fever virus (ASFV) is a highly stable DNA virus showing little genetic variation among genomes. This genetic stability has often posed challenges in tracking variants and identifying potential transmission chains as ASFV spreads into new regions. While mutations within individual sequenced genes are infrequent, the application of whole-genome sequencing enables the identification of neutral and functional mutations across the entire genome, contributing to a deeper understanding of ASFV evolution and epidemiology. In this study, we sequenced whole genomes from 25 ASFV positive samples collected from Vietnam, Timor-Leste and Papua New Guinea (PNG). We classified mutations across ASFV genes and non-coding regions, while identifying mutations that may act as candidates for geographic-based population genetic structuring. Overall, ASFV samples showed >99.8% genetic similarity to the Georgia 2007 isolate reference genome. Nonetheless, emergent genetic clusters rooted in geographic location were apparent. Multiple nonsynonymous mutations were found in sequenced genomes, often unique to ASFV collected within only one studied country. Of the observed mutations, four were found within genes of known function (*E199L*, *CP80R*, *B962L* and *B602L*), with the latter (*B602L*) being a novel mutation within a known targeted marker gene for conventional genotyping of the virus. This work highlights the benefits of incorporating whole-genome sequencing into ongoing ASFV surveillance to better reflect the natural population genetic structuring within the species as it has spread across the Asia–Pacific region. Additionally, this work highlights the value of whole-genome sequencing as a tool for actively monitoring emergent variants displaying functional divergence in key genes associated with ASFV proliferation and host selection.

## 1. Introduction

African swine fever (ASF) is a deadly disease, with the mortality rate of highly virulent isolates approaching 100% in domestic pigs. ASF is caused by infection with ASF virus (ASFV), a large double stranded DNA virus and the sole member of the *Asfivirus* genus, Asfarviridae family [1]. Since first being described in 1921 in Kenya [2], at least 24 distinct genotypes have been defined based on nucleotide differences in the gene coding the capsid protein p72 [3–5]. A recent revision of the classification of ASFV genotypes through p72 classification utilising the predicted protein similarities of the p72 protein places the number of distinct genotypes at six, however [6]. Although all genotypes naturally circulate within sub-Saharan Africa, ASFV has been translocated into Europe twice since 1957, resulting in various outbreaks and global spread [7]. Since the introduction of ASFV Genotype II into Georgia in 2007 [7], it has spread throughout Europe and into the Asia–Pacific, posing a significant and ongoing threat to global domestic pig industries and to small holder farmers in resource poor countries [8].

DNA-encoded viruses, such as ASFV, are known for their remarkable stability, typically exhibiting very slow evolution and mutation rates when compared to RNA-encoded viruses [9]. The stability of the ASFV genome has been underscored by the strong genomic resemblance of current ASFV genomes to those discovered in the genomes of ticks estimated to have been embedded in the tick genome over 1.4 million years ago [10]. Stability across contemporary time scales is evident following the emergence of ASFV in Georgia in 2007. Despite spreading into multiple different nations, the genome has remained largely unchanged compared to the ancestral Georgia/07 sequence [11, 12]. The remarkable genetic preservation of ASFV has facilitated the effective classification of genotypes, primarily relying on a select few specific genes within the genome, namely, *B602L*, *B646L*, *CP204L*, *EP402R*, *E183L* and the intergenic region (IGR) between selected genes, including MGF-505-9R/-10R, *I73R*/*I329L* and *I329L/I215L*, among other highly specific markers shown to delineate structure at a country/neighbouring country scale exclusively [12–16].

The increasing utilisation of whole-genome sequencing has begun to challenge the perception of ASFV being too stable to undergo microevolution on contemporary time scales [17]. Small-scale variants have been increasingly documented, including an accumulation of point mutations across coding and non-coding regions in Germany and Poland, or in India [17, 18]. Furthermore, large-scale structural mutations have been identified including tandem repeat variation amongst sequences within South Korea [19] and multiple synonymous mutations and large (>1 kb) deletions within China [20, 21]. Importantly, of the novel variants detected across countries by whole-genome sequencing methods, few result in changes to genes that are used for genotype classification of ASFV [17, 18, 20, 21].

As ASFV variants are increasingly observed in Europe and Asia through whole-genome sequencing, it raises the question of the extent to which different variants have taken root as ASFV spreads throughout the Asia–Pacific region. In this study, we contribute 24 additional ASFV whole genomes to the growing dataset of sequences collected from the Asia–Pacific. Through sequencing these samples, we determined whether circulating ASFV strains within the countries of Vietnam, Papua New Guinea (PNG) and Timor-Leste have diverged at the whole-genome level to a degree that demonstrates significant population differentiation, resulting in unique geographic signatures for each country. Upon classifying the degree of population differentiation occurring between ASFV in each country, we further identified whether any individual mutations within the genome served as indicators of geographic origin for any of the investigated countries. Last, we classified all mutations by their impact on the expression of known ASFV genes.

## 2. Methods

### 2.1. Sample Collection

**Figure 1:**
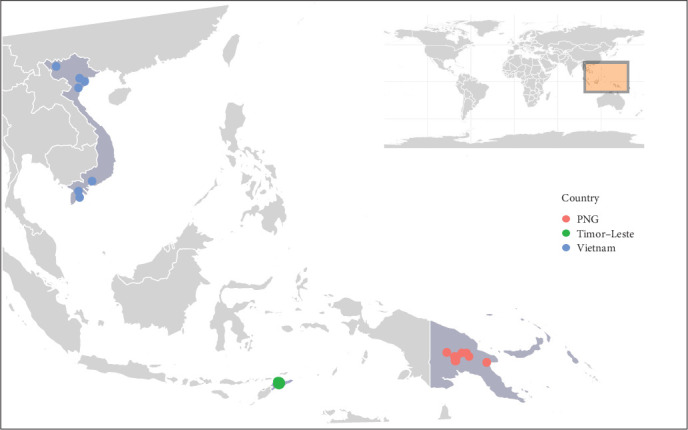
Map of location of ASFV infected pigs in each of the three countries. Countries where samples were collected are highlighted. Collection sites represent the province of collection and not exact co-ordinates.

Samples from 24 ASFV infected domestic pigs were collected from sick and dying pigs as part of disease investigation activities from multiple provinces in Vietnam and PNG and from the Dili District in Timor-Leste. Samples from Vietnam were collected across seven provinces between February and June 2019 following the emergence of ASFV in the country [22]. The two samples from Timor-Leste were collected from Dili municipality in September 2019 during the first outbreak [23], while samples from PNG were collected from six highlands provinces between March and June 2020 following the emergence of ASF in the southern Highlands [24]. Two additional samples collected in March 2023 from an isolated outbreak in the lowlands province of Morobe were also included in the study. The samples comprised a combination of EDTA-whole blood from infected pigs, nasal swabs, tissue samples and tissue homogenate submitted to the Australian Centre for Disease Preparedness for laboratory diagnosis and ASFV genome sequencing (Figure 1 and Table 1). Of the samples collected, 15 were used for direct nucleic acid extraction, while eight were cultured before DNA extraction. A single sample was both cultured and had direct nucleic acid extracted to confirm that minimal nucleotide changes between the two sample types occurred from culturing (Table 1).

### 2.2. DNA Extraction and Sequencing

Total nucleic acid was extracted from each sample using the MagMAX Viral/Pathogen Nucleic Acid extraction kits (Thermo Fisher) following the manufacturer's protocol. Samples were then prepared for next generation sequencing using the Nextera XT DNA Library Preparation Kit following the manufacturer's protocol (Illumina).

To increase the concentration of viral reads in each extraction, after the application of Nextera dual index barcodes to each library, library preparations were enriched using MyBaits hybridisation (Supporting Information 1 for MyBaits probe design) following the manufacturer's protocol (Arbor Biosciences). MyBaits probes were designed by Arbor Biosciences using the China/Anhui/2019 genome (MK128995.1) as a reference. Briefly, nucleic acid material was combined with hybridisation probes and master mix, denatured at 95°C and hybridised at a temperature of 65°C overnight. Following hybridisation, samples were purified through magnetic beads and the sample library was resuspended. Library amplification was then undertaken following the manufacturer's protocol (Arbor Biosciences).

For each prepared sample, fragment size and DNA concentration were estimated using a Bioanalyser 2100 expert high sensitivity DNA kit (Agilent) and a Qubit high sensitivity dsDNA kit (Thermo Fisher), respectively. Based on DNA fragment size and concentration, samples were normalised to equal molarity and pooled prior to sequencing. Each pool was then loaded onto either a Nextseq1000/2000 300 cycle P1 cartridge and sequenced on a Nextseq2000 or a standard Miseq 300 cycle cartridge and sequenced on a Miseq v3.

### 2.3. Generation of ASFV Reference Database

Reference genomes were identified by downloading all highly similar full genomes to the NCBI reference sequence *NC_044959.2*, which is the most recent version of the sequenced Georgia/07 isolate, representing the current Genotype II pandemic strain. The full genome of *NC_044959.2* was used to perform a BLASTn search against all nucleotide sequences (nr/nt database) available within NCBI Genbank web interface using standard settings. We then selected genomes that exhibited both >99% identity and >95% query coverage compared to *NC_044959.2*. However, we implemented several additional criteria for filtering, as follows: genomes were removed if they were labelled as incomplete, >2% of the genome was given in IUPAC ambiguity codes for nucleotide degeneracy or if both of the following criteria were met; the genome showed large indels in the inverted terminal repeat (ITR) region compared with Georgia/07 and was generated exclusively from short read data. Additionally, sequences that showed excessive (20+) mutations in homopolymer regions, which were not present in other reference sequences, were removed due to potential assembly errors. After filtering, 93 reference genomes remained and were carried over into future analyses.

### 2.4. Data Filtering and De Novo Assembly

Raw fastq reads were filtered and adaptor trimmed using fastp v0.23.2 with the following settings: minimum length >75, individual base minimum quality filter of 15, with a 4 bp front sliding window and average minimum quality filter of 20. Filtered reads were aligned against the domestic pig genome (*GCA_000003025.6*) to filter out host reads using bowtie2 v2.4.5 with default settings and very sensitive mode. The non-aligned reads were then assembled into contigs using spades v3.15.5 using default settings and pipeline option ‘—careful' chosen. Assembled contigs were then scaffolded together using a reference-guided scaffolding approach using multi-CSAR (v 1.0) with the 93 NCBI reference genomes guiding the stitching of contigs.

### 2.5. Genome Polishing and Whole Genome Phylogenetic Analysis

As all genomes sequenced here utilised short read sequencing data (excluding MW396979 ASFV/Timor-Leste/2019/1 [25]), reliably generating complete genomes including the ITR regions was not achievable with an exclusively de novo approach. To address this issue, we implemented a two-step de novo and reference aligned approach, where de novo assembled genomes were first used to identify the most closely related NCBI reference genome to act as the reference for assembly.

All genomes assembled through multi-CSAR and the 93 reference genomes were aligned using MAFFT v7.508 with an open and extension penalty of 2 and 0.12, respectively, up to 1000 iterations, and 150 ‘retree' calibrations. A phylogenetic tree was then generated from the alignment using IQ-tree v2.2.0.3 with 1000 ultra-fast bootstraps and the mutation model identified through ModelFinder implemented within IQ-tree.

The most closely related reference genome to each sample was then used to align the host filtered reads of each respective sample using bowtie2 v2.4.5 (with settings previously described). The aligned sam files were then imported into Geneious Prime v2020.2.5, which was used to generate consensus sequences with a read certainty threshold of 65% and N called if read depth was <4. The de novo assemblies of each sequence were then aligned to each generated consensus sequence to identify any indels not detected by a reference guided approach. Consensus sequences were then realigned with downloaded reference sequences using MAFFT v7.508 (with settings as previously described) and expansions on homopolymer sites present in only one sample were removed. Filtered reads were then realigned to the consensus sequences using bowtie2 v2.4.5 (with settings as previously described) for each sample and sam files imported into Geneious Prime v2020.2.5 to generate consensus sequences. At this stage, sites where homopolymers were deleted in the prior alignment were manually inspected to confirm their true presence/absence. All consensus sequences were then aligned with NCBI reference sequences using MAFFT v7.508 (with settings as previously described) and the ITR regions of all genomes were removed. A phylogenetic tree of the trimmed alignment was then generated using IQ-tree with settings previously described but with the addition of 1000 replicates of SH-aLRT comparison tests for significance.

### 2.6. Genome Annotation and Core Gene Analysis

Complete genomes for each sample were annotated in Geneious Prime v2020.2.5 using the ‘Annotate from Database' function with a minimum similarity score of 90% to known annotations in the fully annotated Georgia/07 genome (‘*NC_044959.2*') and only best match annotations found. Core genes found across >95% of references and samples were then extracted and genes were individually aligned using MAFFT v7.508 (with settings as previously described). A combined core gene tree was then generated using IQ-tree with settings previously described but with independent mutation models per gene.

A subset comprising only commonly utilised marker genes (*B602L*, *B646L*, *CP204L*, *EP402R*, *E183L* and the tandem repeat sequence (TRS) between *I73R* and *I329L* (*TRS*)) was additionally extracted. These markers were then aligned using MAFFT v7.508 (with settings as previously described) and a phylogenetic tree was generated using IQ-tree (with settings previously described).

### 2.7. Identification of Geography Based Phylogenetic Signatures

The maximum likelihood trees generated by IQ-tree were imported into R v4.2.2 and were plotted using the ggtree package v3.7.1.00 [26], with reference sequences forming large clades having all except two sequences removed from the figure to allow for clearer interpretation of study results. Reference alignments were imported into R using the seqinr package v4.2.23 [27] and SNPs/indels diverged from Georgia/07 were identified using a combination of base R and the package tidyr v1.2.1 [28] (see data accessibility). Individual SNPs/indels were then tallied within and across specific countries to calculate the presence of mutations found in a single country and whether the mutation was fixed across all samples within that country. For the core gene alignments, the R package Biostrings v2.64.1 [29] was used to translate gene sequences and mutations within genes were classified based on whether they were synonymous or nonsynonymous. Country-specific mutations were then plotted alongside generated phylogenetic trees.

## 3. Results

### 3.1. Complete Genomes ASFV Sequences

Whole-genome sequencing was completed on 24 ASFV samples from three countries (Table 1) with one sample being sequenced from both original tissue and derived isolate. The length of complete genomes varied from 190,296 nt (19-02791-05/Vietnam/2019 and 19-02791-07/Vietnam/2019) to 192,238 nt (*MW396979* ASFV/Timor-Leste/2019/1). The average read depth varied from 25x to 9019x. Notably, only four of the genomes had 0.01% or higher bases called as N for missing data (Table 2). The completed genomes of the original sample and isolate sample, where both were sequenced (20-01180-06/PNG/2020 and 20-01180-06-02/PNG/2020), showed 100% nucleotide similarity. All subsequent statistics are reported excluding the original sample (20-01180-06/PNG/2020), so as not to double count this genome.

### 3.2. Conventional Genotyping Marker Subset

Four common marker genes used for ASFV molecular typing were extracted from the whole genomes, the structural protein p72 (*B646L*), the IGR between the *I73R* and *I329L* genes, the central variable region (CVR) of the *B602L* gene and the protein CD2v (EP402R) [12–14, 30]. Across all samples, the p72 genotype was identified as II, the IGR type as II, the CD2v serogroup as 8 and for all samples excluding 20-01991-11/PNG/2020 and 20-01992-0061/PNG/2020 the CVR subgroup was XXXII/CVR1 (Supporting Information 2). For samples 20-01991-11/PNG/2020 and 20-01992-0061/PNG/2020, a 12 nucleotide deletion was observed, encoding the tetramer repeat sequence of BNDBNDBAL, which has no associated CVR subgroup classification [5].

### 3.3. Whole Genome Analysis of ASFV

**Figure 2:**
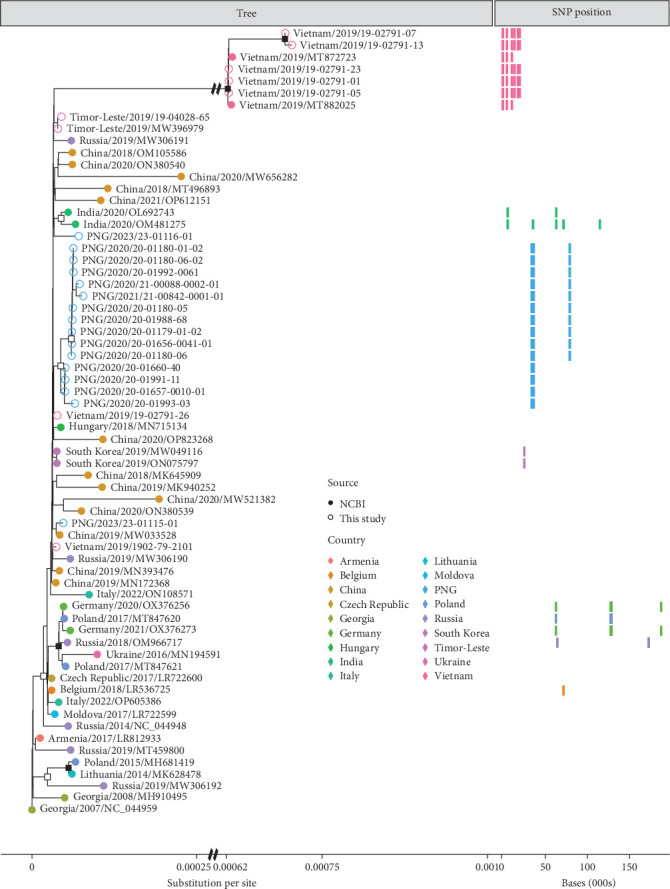
Maximum likelihood phylogenetic tree of ASFV genomes sequenced in this study based on alignment with a proportional subset of reference sequences taken from NCBI Genbank. The left side displays the phylogenetic tree developed with the reference Georgia/07 sequence used as the root. The right side displays the location of all SNP point mutations within the genome, which are found to be present exclusively in two or fewer countries and at a frequency above 75% within a country. The left scale displays the number of substitutions per site, while the right scale shows the nucleotide base position of each SNP relative to the Georgia/07 reference genome. The tree was generated using a HKY+F+I mutation model. Nodes with an open square displayed >80% confidence from SH-aLRT and ultra-fast bootstrap supports, while nodes with a closed square displayed >95% confidence from SH-aLRT and ultra-fast bootstrap supports. Sequences generated in this study are denoted with an open circle while NCBI reference sequences are denoted with a closed circle.

We observed subtle yet discernible variation from the Georgia/07 reference sequence (*NC_044959.2*) in ASFV genomes sequenced from PNG, Timor-Leste and Vietnam (Figure 2). Within each of these three countries, most samples clustered together, forming distinct and country-specific monophyletic clades (Figure 2). Notably, two PNG samples from 2023 (23-01115-01 and 23-01116-01) clustered together but separately from all other PNG samples. Additionally, two samples from Vietnam in 2019 (19-02791-26 and 1902-79-2101) did not exhibit a clear clustering pattern with any specific clade or with other Vietnam samples.

In total, among all samples sequenced here, 2748 nucleotide differences were found in comparison to the Georgia/07 reference genome. These differences were the result of 523 distinct mutations scattered across the ASFV genome (Supporting Information 3). Among these mutations, 22 were identified as insertions, 331 as deletions, and 170 as substitutions (Supporting Information 3).

Most Vietnam samples showed nearly fivefold mutational differences from the Georgia reference than any other sample (Figure 2). Of the observed 523 unique mutations across all samples, 411 were found exclusively within the first 1000 base pairs of Vietnam ASFV genomes (excluding the ITR region) making up 2060 of the 2748 nucleotide differences found within this study, indicating the 5′ end of the ASFV genomes from Vietnam are strongly diverged from Georgia/07 (Supporting Information 3). For all samples from Vietnam that showed high mutational differences from the Georgia reference, the read depth over this diverged region was >300.

No mutations were fixed in all samples from either PNG or Vietnam. However, this is the product of some individual genomes analysed that did not fall into the identified country-specific monophyletic clades, rather than variation of mutations present within the clades themselves. This is most clearly seen in five of the Vietnam samples (1902-791-01, 1902-791-05, 1902-791-07, 1902-791-13 and 1902-791-23), where all 409 mutations at the 5′ end of the genome were present in all five samples. Three mutations were found across both samples taken from Timor-Leste, but were not found in any ASFV genomes from other countries; however, given that only two genomes from Timor-Leste were analysed, these may not be truly fixed within the country. Four mutations were found in over 75% of ASFV samples from PNG, while not being present in any other country (Supporting Information 3). Of the mutations found exclusively within Vietnam, only three were present in over 75% of genomes (Supporting Information 3). This comparatively low number reflected the dichotomy of genetic structuring seen within Vietnam and PNG samples, with two samples forming distinct clusters in each country (1902-79-2101 and 1902-791-26 (Vietnam) and 23-01116-01 and 23-01115-01 (PNG); Figure 2).

### 3.4. Comparison of ASFV Core Genes

**Figure 3:**
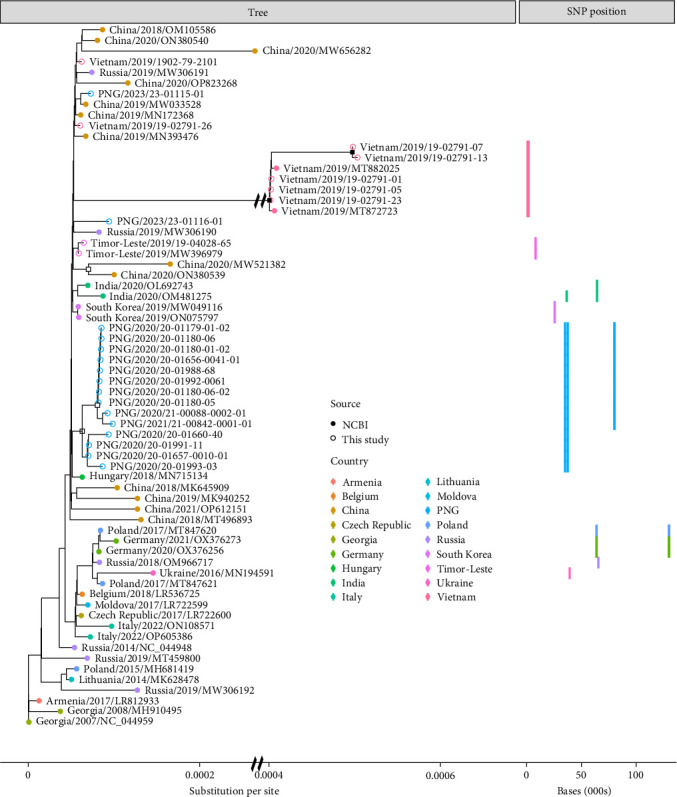
Maximum likelihood phylogenetic tree of all core gene regions of sequenced ASFV genomes from this study and reference sequences taken from NCBI Genbank. The left side displays the phylogenetic tree developed with the reference Georgia/07 sequence used as the root. The right side displays the location of all SNP point mutations within the genome which are found to be present exclusively in two or fewer countries and at a frequency above 75% within a country. The left scale displays the number of substitutions per site, while the right scale shows the nucleotide base position of each SNP relative to the Georgia/07 reference genome. The tree was generated using a HKY+F+I mutation model. Nodes with an open square displayed >80% confidence from SH-aLRT and ultra-fast bootstrap supports, while nodes with a closed square displayed >95% confidence from SH-aLRT and ultra-fast bootstrap supports. Sequences generated in this study are denoted with an open circle while NCBI reference sequences are denoted with a closed circle.

One hundred eighty-five genes/open reading frames (ORFs) were identified across >95% of ASFV genomes (Supporting Information 4). The maximum likelihood tree of all core genes showed less overall genetic differentiation compared with the full genome analysis, with all except Vietnam samples showing an approximately 40% lower nucleotide substitution rate (Figures 2 and 3).

The gene specific tree revealed that Timor-Leste samples formed a monophyletic clade, while most samples from PNG (13/15) and Vietnam (5/7) formed respective monophyletic clades (Figure 3). Four mutations were present in most PNG samples (>75% of samples), while not being present in samples from any other country (Table 3 and Supporting Information 5). No mutations were present in >75% of Vietnam samples due to samples 1902-79-2101 and 19-02791-26 showing near zero genetic differentiation from the Georgia/07 reference sequence. Nonetheless, 32 mutations were present in five of the seven Vietnam samples sequenced here and both Vietnam reference sequences (Table 3 and Supporting Information 5). Four mutations were found in both samples from Timor-Leste and which were found in no other genomes from any other country (Table 3 and Supporting Information 5).

### 3.5. Mutations Within ORF Regions

In the core genes across all sequenced samples, a total of 647 nucleotides were identified as differing from the Georgia/07 reference genome. These 647 nucleotide differences can be categorised into 82 mutations at distinct sites along the ASFV genome. Of these mutations, 41 were found within the gene *MGF360-1La* and 15 within gene *MGF360-21R*. All 56 of the mutations observed in the above genes were found exclusively within Vietnam samples, 41 of which resulted in amino acid substitutions, 12 were silent mutations, two were insertions of three bases and one was a deletion of 21 bases near the end of the gene. The remaining 26 mutations were found across 21 genes and were classified as eight silent mutations, 14 missense substitutions, one insertion, one deletion, one nonsense substitution and one insertion within a homopolymer region (Supporting Information 5 and Table 3). One mutation in the *E199L* gene was the only mutation found across two countries within this study (PNG and Vietnam). Of the total 23 mutated genes observed, four led to changes in the translation of genes of known function (*B602L*, *B962L*, *CP80R* and *E199L*; Table 3).

## 4. Discussion

Here, we characterised the whole genomes of 24 ASFV genomes from infected domestic pig samples collected across three different countries, Vietnam, Timor-Leste and PNG. Although double stranded DNA viruses are comparatively highly genetically stable [9], we have identified multiple nonsynonymous mutations in the genomes of ASFV sampled from each of the countries studied herein. Importantly, of all the mutations observed, only one was within a gene region commonly sequenced for ASFV genotyping and isolate characterisation (*B602L*) and was only found in two of the 15 samples sequenced from PNG, highlighting the importance of whole-genome sequencing in characterising ASFV microevolution. While many mutations were detected exclusively within only one country, none were found to reliably delineate the country of origin for all samples from that country. Moreover, four mutations were found to result in nonsynonymous changes within genes of known function in the ASFV replication cycle (*E199L*, *CP80R*, *B962L* and *B602L*), providing further support for the importance of ongoing whole genome surveillance to detect emergent variants of concern. This work provides additional evidence of the dynamic nature of ASFV, highlighting the virus's potential to evolve across contemporary timeframes.

### 4.1. Use of Targeted Mutations for ASFV Tracing and Evolution

Regionalised genetic structuring of ASFV has been documented to varying extents utilising a number of variable and known gene regions/markers within the ASFV genome including *B602L*, *B646L*, *E183L*, *CP204L*, *EP402R* or the TRS region between *I73R* and *I329L*, among others [12–16, 30]. Of the above markers, the TRS region is the most effective at differentiating sub lineages of ASFV Genotype II in the Asia–Pacific [18, 19, 30, 32], yet across all the listed key gene regions, every sample sequence was identical to the majority of reference sequences from Asia and Europe. The only exception to this was two samples from PNG (12.5% of those sequenced from PNG), which showed variation in the gene *B602L*. This variation was a deletion of a repeat region defined as a repeat tetramer in the CVR and has not been detected previously [15]. Greater success in elucidating regional genetic structuring has been found using whole genome comparisons, with mutations being detected in multiple regions of the ASFV genome distinct to specific localities [17, 18, 20].

Clear geographic and genetic structuring was observed at a whole genome level within ASFV genomes from PNG, Vietnam and Timor-Leste. Further, this pattern was observed from both whole genomes and core gene phylogenies suggesting the emerging geographic and genetic structuring is not exclusively driven by non-coding regions of the genome. This pattern of structuring was the result of neutral, missense and nonsense point mutations and larger insertions and deletions (Table 3). Despite the clear structuring seen at a whole genome scale, some samples collected from both PNG and Vietnam did not cluster with others from their respective country of collection. Although consistent population genetic structuring has been observed in geographical regions in Europe [17, 33, 34], the lack of genetic clustering of all samples within each country is more indicative of what has been seen in Russia, China and East Asia [35, 36]. The presence of distinct clades in each country may suggest that there are multiple genetic clusters circulating or that the sequences are the result of secondary introductions. Given the complexity of pig value chains and the density of wild boar within many countries in the Asia–Pacific, there is a sustained probability of repeat secondary introductions occurring within countries [37, 38]. Such introductions are likely to be a combination of both legal and illegal movements of pork and live pigs and through wild boar movements. For example, illegal crossings have resulted in secondary introductions into northern Vietnam, although whether these were the result of live pigs or pork products is unclear [30, 39], while routine monitoring of food waste and pork products consistently returns evidence of ASFV at major shipping ports [40, 41]. With multiple potential routes of reintroduction of the virus, there is a possibility of variants being repeatedly introduced and either replacing specific localised variants or reducing their frequency within a country. Further, given the recent detection of highly virulent Genotypes I and II recombinants [42], the potential of secondary introductions may increase the chances of coinfections from distinct genotypes, and therefore, the possibility of emergent recombinant genotypes within the studied countries.

When considering the possibility of genomic variants circulating within countries being composed of a primary introduction and one or more secondary introductions, relying on fixed SNPs for regional classification is likely to return few regionalised SNPs in general and potentially no regionalised SNPs for countries which have experienced multiple introductions. Despite the possibility of reintroductions, there is still potential for whole genome phylogenies to reliably infer the likely epidemiological origin of localised ASFV spread, as already reported for ASFV as it has spread across Asia [35, 43]. Nonetheless, the nature of outlier genomic sequences shows that for ASFV genomic tracking to be most effective, more whole-genome sequencing is required to classify all circulating microvariants present within countries.

### 4.2. Potential Functional Significance of Mutations Found

Among the genomes of ASFV sequenced here, mutations were observed within 23 known genes/gene families, with 18 genes showing changes to translation. Of the 18 genes with a change in translation, nine were within multigene families and a further five were of unknown function [4, 31]. In prior investigations of ASFV Genotype II mutation rates, rapidly emerging mutations have been associated with initial mutations within key DNA repair genes (e.g., *NP419L* and *O174L* genes) [17, 20]. Despite observing nearly 3000 nucleotide changes from the Georgia/07 reference genome among all 24 sequenced samples, no mutations were detected in any DNA repair genes.

The most variable gene region was the *MGF360-1L* multigene family member, with five of the seven genomes collected from Vietnam showing dozens of point mutations and a premature termination of the gene. Although the function of these gene families is not known definitively, the *MGF-360* gene family may play a role in virulence, with deletions resulting in reduced virulence in pigs [44–46]. Specifically, mutations in *MGF360-1L* may influence host range and host cell survival [46, 47]. Of the four genes of known function found to have a nonsynonymous mutation, each plays an important role in ASFV function. *E199L* has been shown to have multiple roles in host cell autophagy and modulating ASFV entry into cells [48, 49], *CP80R* encodes RNA polymerase subunit 10 [4], *B962L* exhibits a role in RNA helicase activity [4] and *B602L* is essential for the formation of the ASFV capsid [50]. The nonsynonymous mutation in *E199L* was only observed in a cultured isolate suggesting that this change did not significantly affect replication. However, subtle effects in replicative fitness would need to be investigated further along with the role of each specific gene mutation.

## 5. Conclusions

Whole-genome sequencing has uncovered country level genetic structuring within ASFV samples taken from PNG, Vietnam and Timor-Leste. While each country exhibited a distinct monophyletic clade, clear outlier samples from PNG and Vietnam were also evident. Moreover, while several mutations were consistently found within all samples of each of the defined monophyletic clades, these mutations were absent in any outlier sequences. This suggests that individual mutations do not reflect the diversity of variants in each country, nor can they consistently classify all sequences to or from a specific point of origin. Importantly, whether the outlier ASFV samples observed in PNG and Vietnam reflect circulating diversity or ongoing reintroductions of variants from neighbouring countries remains a pressing yet unanswered question. Last, nonsynonymous mutations were observed in at least one sample from every country, despite the relatively short timeframes ASFV has been circulating in the Asia–Pacific. This underscores the importance of continuous whole genome classification to identify emerging variants of concern within the region and to track the evolution of ASFV as it continues to circulate and spread in regions of Asia and the Pacific.

## Figures and Tables

**Table 1:** Details of samples utilised to generate ASFV genomes.

Sample name	Collection date	Country	Province	District	Sample type
19-02791-01/Vietnam/2019	14/02/2019	Vietnam	Thai Binh	Hung Ha	Tissue homogenate
19-02791-05/Vietnam/2019	17/05/2019	Vietnam	Ha Noi	Thanh Oai	Tissue homogenate
19-02791-07/Vietnam/2019	14/05/2019	Vietnam	Dien Bien	Tua Chua	Tissue homogenate
19-02791-13/Vietnam/2019	24/04/2019	Vietnam	Dong Nai	Trang Bom	Tissue homogenate
19-02791-23/Vietnam/2019	4/06/2019	Vietnam	Bac Lieu	Vinh Loi	Tissue homogenate
19-02791-26/Vietnam/2019	4/06/2019	Vietnam	Hau Giang	Chau Thanh A	Tissue homogenate
1902-79-2101/Vietnam/2019^a^	23/02/2019	Vietnam	Thanh Hoa	Yen Dinh	Isolate derived from tissue homogenate
ASFV/Timor-Leste/2019/1 [25]^a^	X/09/2019	Timor Leste	N/A	Dili District	Nasal swab
19-04028-65/Timor-Leste/2019	X/09/2019	Timor Leste	N/A	Dili District	Nasal swab
20-01179-01-02/PNG/2020^a^	12/03/2020	PNG	Southern Highlands	Tende Village	Isolate derived from nasal swab
20-01180-01-02/PNG/2020^a^	12/03/2020	PNG	Southern Highlands	Tupiri Village	Isolate derived from serum
20-01180-05/PNG/2020	12/03/2020	PNG	Southern Highlands	Tupiri Village	Tissue-spleen
20-01180-06-02/PNG/2020^b^	12/03/2020	PNG	Southern Highlands	Tupiri Village	Isolate derived from tissue-lymph node
20-01180-06/PNG/2020^b^	12/03/2020	PNG	Southern Highlands	Tupiri Village	Tissue-lymph node
20-01657-0010-01/PNG/2020^a^	15/04/2020	PNG	Southern Highlands	Mendi/Nipa	Isolate derived from whole blood (EDTA)
20-01660-40/PNG/2020	15/04/2020	PNG	Enga	N/A	Whole blood (EDTA)
20-01988-68/PNG/2020	3/06/2020	PNG	Hela	N/A	Whole blood (EDTA)
20-01991-11/PNG/2020	27/05/2020	PNG	Western Highlands	Mt Hagen	Whole blood (EDTA)
20-01992-0061/PNG/2020	3/06/2020	PNG	Enga	N/A	Whole blood (EDTA)
20-01993-03/PNG/2020	27/05/2020	PNG	Southern Highlands	Mendi	Whole blood (EDTA)
20-01656-0041-01/PNG/2020^a^	15/04/2020	PNG	Hela	N/A	Isolate derived from whole blood (EDTA)
21-00088-0002-01/PNG/2020^a^	2/12/2020	PNG	Jiwaka	N/A	Isolate derived from whole blood (EDTA)
21-00842-0001-01/PNG/2021^a^	7/02/2021	PNG	Chimbu	Chuave	Isolate derived from whole blood (EDTA)
23-01115-01/PNG/2023	2/03/2023	PNG	Morobe	Wampar LLG	Whole blood
23-01116-01/PNG/2023	2/03/2023	PNG	Morobe	Wampar LLG	Whole blood

*Note*: Each sample name contains a unique identifier code, country and year of collection.

^a^Sample was a viral isolate grown from the initial material.

^b^Samples represent an initial sample and a secondary isolate derived from the sample.

**Table 2:** Summary of ASFV genomes sequenced and assembled in this study.

Isolate name	Genome length (nt)	GC content (%)	Total reads aligned	Read depth median (±standard deviation)	Percent of genome <20 read depth (%)	Percent missing bases (%)	Accession number	Total nucleotide differences from Georgia/07
19-02791-01/Vietnam/2019	190,301	38.4	2,421,032	1270 (±1524.87)	<0.01	<0.01	PV592812	443
19-02791-05/Vietnam/2019	190,298	38.4	2,049,063	1395 (±564.41)	<0.01	<0.01	PV592813	442
19-02791-07/Vietnam/2019	190,301	38.4	5,659,062	3380 (±2737.01)	<0.01	<0.01	PV592813	459
19-02791-13/Vietnam/2019	190,299	38.4	2,744,352	2053 (±880.22)	0.12	<0.01	PV592815	465
19-02791-23/Vietnam/2019	190,307	38.4	792,493	578 (±237.17)	<0.01	<0.01	PV592816	445
19-02791-26/Vietnam/2019	190,581	38.4	4,800,005	3520 (±1263.89)	<0.01	<0.01	PV592817	22
1902-79-2101/Vietnam/2019^a^	190,574	38.4	2,181,502	1537 (±566.44)	<0.01	<0.01	PV592818	22
ASFV/Timor-Leste/2019/1 [25]^a^	192,238	38.4	108,132	72 (±30.80)	2.66	0.1	MW396979	24
19-04028-65/Timor-Leste/2019	192,237	38.4	34,319	25 (±12.47)	34.6	0.3	PV592811	22
20-01179-01-02/PNG/2020^a^	190,584	38.4	7,829,773	5820 (±2157.3)	<0.01	<0.01	PV592796	24
20-01180-01-02/PNG/2020^a^	190,580	38.4	3,990,842	2858 (±1088.19)	<0.01	<0.01	PV592797	22
20-01180-05/PNG/2020	190,596	38.4	2,364,370	1638 (±802.29)	0.05	<0.01	PV592798	22
20-01180-06-02/PNG/2020^a,b^	190,590	38.4	12,807,240	9019 (±3591.31)	<0.01	<0.01	PV592799	22
20-01180-06/PNG/2020^b^	190,590	38.4	892,647	604 (±493.25)	0.01	<0.01	NA	22
20-01657-0010-01/PNG/2020^a^	190,581	38.4	1,416,840	1035 (±394.01)	<0.01	<0.01	PV592801	23
20-01660-40/PNG/2020	190,583	38.4	693,792	487 (±270.51)	0.19	0.01	PV592802	32
20-01988-68/PNG/2020	190,589	38.4	401,202	299 (±135.18)	0.01	<0.01	PV592803	23
20-01991-11/PNG/2020	190,582	38.4	286,004	213 (±111.84)	0.39	0.1	PV592804	35
20-01992-0061/PNG/2020	190,594	38.4	1,057,217	790 (±306.1)	0.03	<0.01	PV592805	23
20-01993-03/PNG/2020	190,590	38.4	4,149,160	2718 (±1715.93)	<0.01	<0.01	PV592806	24
20-01656-0041-01/PNG/2020^a^	190,590	38.4	2,088,621	1519 (±631.06)	<0.01	<0.01	PV592800	24
21-00088-0002-01/PNG/2020^a^	190,582	38.4	4,782,765	3361 (±1329.67)	<0.01	<0.01	PV592807	25
21-00842-0001-01/PNG/2021^a^	190,586	38.4	3,966,583	2735 (±1111.67)	<0.01	<0.01	PV592808	26
23-01115-01/PNG/2023	190,586	38.4	296,765	409 (±379.26)	0.86	0.1	PV592809	23
23-01116-01/PNG/2023	190,586	38.4	1,068,335	809 (±481.23)	0.05	<0.01	PV592810	29

^a^Sample was a viral isolate grown from the initial material.

^b^Samples represent an initial sample and a secondary isolate derived from the sample.

**Table 3:** Presence of gene mutations within ASFV genomes from samples collected in PNG, Timor-Leste and Vietnam using the ASFV Georgia/07 genome as a reference sequence.

Gene	Function [4, 31]	PNG	Timor-Leste	Vietnam	Mutation type	Mutation position in gene
ASFV G ACD 00240	Unknown	Present (11)	Conserved	Conserved	Frameshift insertion	57
ASFV G ACD 01020	Unknown	Present (2)	Conserved	Conserved	Silent	84
B407L	Unknown	Present (1)	Conserved	Conserved	Silent	873
B602L	Chaperone protein of p72 (folding of capsid)	Present (2)	Conserved	Conserved	In frame deletion	558–569
B962L	Helicase superfamily II	Present (1)	Conserved	Conserved	Missense substitution	2164
CP80R	Replication: RNA polymerase subunit 10	Present (1)	Conserved	Conserved	Missense substitution	133
E199L	J18L transmembrane domain	Present (2)	Conserved	Present (1)	Missense substitution(s)	245 (Vietnam), 373, 376 (PNG)
I177L	Unknown	Present (1)	Conserved	Conserved	Silent	435
I226R	Unknown	Conserved	Conserved	Present (1)	Missense substitution	202
I7L	Unknown	Present (4)	Conserved	Conserved	Missense substitution	275
M1249L	Unknown	Present (2)	Conserved	Conserved	Missense substitution, silent substitutions	661, 682, 691
M448R	Unknown	Present (8)	Conserved	Conserved	Missense substitution	161–163
MGF110-4L	Multigene (contains KDEL ER retrieval sequence and transmembrane domain)	Conserved	Present (2)	Conserved	Missense substitution	325
MGF300-4L	Multigene	Present (2)	Conserved	Conserved	Silent substitution, nonsense substitution	143, 648
MGF360-12L	Multigene	Present (1)	Conserved	Conserved	Missense substitution	699
MGF360-14L	Multigene	Conserved	Present (2)	Present (6)	Frameshift insertion (homopolymer)	846
MGF360-16R	Multigene	Present (1)	Conserved	Conserved	Missense substitution	22
MGF360-1La	Multigene	Conserved	Conserved	Present (5)	Insertions, missense substitution, silent	472, 478, 488, 496, 505, 507, 556, 558, 570, 577, 580, 630, 640, 647, 653, 661, 670, 673, 675, 683, 703, 706, 708, 709, 711, 778, 798, 809, 812 (Missense) 482, 692 (Insertion) 513, 543, 573, 591, 645, 678, 599, 753, 760 (Silent) 819 (Nonsense)
MGF360-21R	Multigene	Conserved	Conserved	Present (2)	Missense substitution, silent	576, 615, 646, 649, 653, 661, 663, 665, 679, 681, 688, 699 (Missense) 634, 636, 660 (Silent)
MGF505-10R	Multigene	Present (2)	Conserved	Conserved	Missense substitution	1580
MGF505-3R	Multigene	Present (13)	Conserved	Conserved	Missense substitution	166
MGF505-5R	Multigene	Present (13)	Conserved	Conserved	Silent	9
NP868R	Guanylyl transferase	Present (1)	Conserved	Conserved	Silent	1367

*Note*: The number of samples that each mutation was found in is shown in parentheses. For additional details of samples, nucleotide base substitutions and positions within each gene, see Supporting Information 5.

## Data Availability

All scripts utilised in the analysis of data associated with this study can be found at https://github.com/James-ODwyer/ASFV_analysis_scripts. All ASFV genomes generated as part of this work have been uploaded to NCBI under accessions PV592796–PV592818. All mutation count data within this study can be found in Supporting Information SIII and SV. The raw FASTQ sequences used to generate the ASFV genomes from each sample have been uploaded to NCBI Sequence Read Archive under the accessions SRR33513695–SRR33513714.

## Supporting Information

Supporting Information 1 Fasta file of Mybaits enrichment probes designed to enrich African swine fever virus genomic DNA.

Supporting Information 2 Microsoft Word document containing molecular typing results for African swine fever virus genomes under commonly used classification gene markers.

Supporting Information 3 Microsoft Excel document containing a list of all mutations across the whole genomes of all African swine fever virus genomes sequenced.

Supporting Information 4 Microsoft Excel document containing a list of all genes, whether they were included in the core-gene phylogeny, the length of each gene, and their start position relative to the Georgia 2007 ASFV reference genome.

Supporting Information 5 Microsoft Excel document containing a list of all mutations within any gene regions for all African swine fever virus genomes sequenced along with the translational impact of each mutation.
