# Supplementary material for: Emergence of Microvariants of African Swine Fever Virus Genotype II in the Asia–Pacific
Source: Transbound Emerg Dis. 2025 Jun 20;2025:9990044. doi: 10.1155/tbed/9990044 (PMC12204745; doi:10.1155/tbed/9990044)
Supplement: Supporting Information 2 — Microsoft Word document containing molecular typing results for African swine fever virus genomes under commonly used classification gene markers. [file 9990044.f2.docx]

**Supplementary material 2. Summary of the analysis of common ASFV marker genes extracted from ASFV whole genomes.**

**Table 1.** Summary table of the molecular typing results for African Swine fever virus using four commonly used marker regions: the structural protein p72 (*B646L), the* intergenic region (IGR) between the *I73R* and *I329L* genes, the central variable region (CVR) of the *B602L* gene and the protein CD2v (*EP402R*).

| Sample | P72 Genotype | IGR Type | Cd2v serogroup | CVR Subgroup type |
| --- | --- | --- | --- | --- |
| 19-02791-01/Vietnam/2019 | II | II | 8 | XXXII/CVR1 |
| 19-02791-05/Vietnam/2019 | II | II | 8 | XXXII/CVR1 |
| 19-02791-07/Vietnam/2019 | II | II | 8 | XXXII/CVR1 |
| 19-02791-13/Vietnam/2019 | II | II | 8 | XXXII/CVR1 |
| 19-02791-23/Vietnam/2019 | II | II | 8 | XXXII/CVR1 |
| 19-02791-26/Vietnam/2019 | II | II | 8 | XXXII/CVR1 |
| 1902-79-2101/Vietnam/2019 | II | II | 8 | XXXII/CVR1 |
| ASFV/Timor-Leste/2019^1^ | II | II | 8 | XXXII/CVR1 |
| 19-04028-65/ Timor-Leste/2019 | II | II | 8* | XXXII/CVR1 |
| 20-01179-01-02/PNG/2020 | II | II | 8 | XXXII/CVR1 |
| 20-01180-01-02/PNG/2020 | II | II | 8 | XXXII/CVR1 |
| 20-01180-05/PNG/2020 | II | II | 8 | XXXII/CVR1 |
| 20-01180-06-02/PNG/2020 | II | II | 8 | XXXII/CVR1 |
| 20-01180-06/PNG/2020 | II | II | 8 | XXXII/CVR1 |
| 20-01657-0010-01/PNG/2020 | II | II | 8 | XXXII/CVR1 |
| 20-01660-40/PNG/2020 | II | II | 8 | XXXII/CVR1 |
| 20-01988-68/PNG/2020 | II | II | 8 | XXXII/CVR1 |
| 20-01991-11/PNG/2020 | II | II | 8 | †undefined |
| 20-01992-0061/PNG/2020 | II | II | 8 | †undefined |
| 20-01993-03/PNG/2020 | II | II | 8 | XXXII/CVR1 |
| 20-01656-0041-01/PNG/2020 | II | II | 8 | XXXII/CVR1 |
| 21-00088-0002-01/PNG/2020 | II | II | 8 | XXXII/CVR1 |
| 21-00842-0001-01/PNG/2021 | II | II | 8 | XXXII/CVR1 |
| 23-01115-01/PNG/2023 | II | II | 8 | XXXII/CVR1 |
| 23-01116-01/PNG/2023 | II | II | 8 | XXXII/CVR1 |

* An incomplete gene region was sequenced for this sample (279/326 nt present), however, excluding ambiguous regions, this sequence showed 100% match to serogroup 8.
† A previously undefined CVR subgroup was detected for these samples, comprising a coded tetramer repeat sequence of BNDBNDBAL, which does not have an assigned subgroup (Nix et al., 2006; Quembo et al., 2018).

Nix, R. J., Gallardo, C., Hutchings, G., Blanco, E., & Dixon, L. K. (2006). Molecular epidemiology of African swine fever virus studied by analysis of four variable genome regions. *Archives of Virology*, *151*(12), 2475–2494. https://doi.org/10.1007/s00705-006-0794-z

Quembo, C. J., Jori, F., Vosloo, W., & Heath, L. (2018). Genetic characterization of African swine fever virus isolates from soft ticks at the wildlife/domestic interface in Mozambique and identification of a novel genotype. *Transboundary and Emerging Diseases*, *65*(2), 420–431. https://doi.org/10.1111/tbed.12700
